# LEO satellite assisted UAV distribution using combinatorial bandit with fairness and budget constraints

**DOI:** 10.1371/journal.pone.0290432

**Published:** 2023-08-23

**Authors:** Ehab Mahmoud Mohamed

**Affiliations:** Department of Electrical Engineering, College of Engineering in Wadi Addwasir, Prince Sattam Bin Abdulaziz University, Wadi Addwasir, Saudi Arabia; TU Wien: Technische Universitat Wien, AUSTRIA

## Abstract

In this paper, an integration between a low earth orbit satellite (LEO-Sat) and unmanned aerial vehicle (UAV) is proposed to assist users in post-disaster areas. In this scenario, multiple UAVs will be distributed to fully cover the victims and provide rescue services, while LEO-Sat provides backhaul links for UAVs to the ground base station (GBS). In this regard, we consider the problem of efficient UAVs distribution to maximize the total sum rate of the victims while assuring fairness in their coverage within the limited resources of UAVs batteries and LEO-Sat bandwidth. In this paper, UAV distribution problem is considered as a combinatorial multi-armed bandit (MAB) with arms’ fairness and limited UAVs battery budget (CMAB-FB) constraints. Additionally, the utilization of LEO-Sat bandwidth resources is optimized based on the average traffic demands of the LEO-UAV links by means of gradient decent algorithm. The results of numerical analysis indicate that the proposed approach outperforms other naïve ben chmarks.

## I. Introduction

In recent years, unmanned aerial vehicles (UAVs) have been increasingly utilized in post-disaster relief efforts, due to their ability to reach remote and hard-to-access areas quickly [1,2]. In post-disaster scenarios, UAVs can provide wireless platforms for user equipments (UEs) belonging to victims and rescue workers. However, effective deployment of UAVs in such scenarios is a complex task that requires balancing multiple conflicting objectives, including coverage, limited UAVs battery life, and communication capabilities [1,2]. The limited transmission range of UAVs may hinder them from communicating directly with the nearest ground base station (GBS) or with each other, impacting their deployment and data gathering capabilities in post-disaster areas. On the other side, low earth orbit satellites (LEO-Sat) have revolutionized wireless communications, offering a myriad of applications that have transformed the way we connect and communicate [3]. LEO-Sats, positioned at an altitude ranging from 500 to 2,000 kilometers above the earth’s surface, provide several advantages in wireless communications [3]. Firstly, their proximity to the earth enables lower latency, reducing signal delays and enhancing real-time communication. Additionally, LEO-Sats offer high bandwidth capabilities, enabling faster data transmission and accommodating the increasing demand for high-speed internet access. These satellites have opened a world of possibilities for wireless communication applications, including global broadband internet coverage, internet-of-things (IoT) connectivity, remote sensing, and disaster management [3]. With their extensive coverage, LEO-Sat provides connectivity to even the most remote areas, bridging the digital divide and facilitating global communication networks. Moreover, they play a crucial role in disaster response, offering immediate connectivity and enabling effective coordination and communication during emergencies [3].

In this paper, an integration between LEO-Sat and UAVs in post-disaster scenario is proposed with the aim of efficiently distributing UAVs among the post-disaster zones. In the proposed scenario, LEO-Sat will be used to provide crucial backhaul links for UAVs, allowing them to communicate with the nearest survival GBS and relay critical data/control information, while considering the limited bandwidth resources of LEO-Sat. This cannot be achieved in traditional scenarios, i.e., without LEO-Sat, due to the destroyed/malfunctioned GBS, and the difficulty for the UAVs to directly connect with the nearest survival GBS due to their limited coverage area. In post-disaster zones, the deployment of UAVs should prioritize maximizing their achievable data rates while considering their limited battery resources and the limited bandwidth resources of LEO-Sat as well as ensuring fairness in coverage among post-disaster zones based on their UEs density. Essentially, subareas with higher UE densities should receive more frequent service than those with lower densities. The challenge of this optimization problem comes from three main folds: 1) How to ensure coverage fairness among UEs in the post-disaster area where the wireless infrastructure is completely destroyed. 2) UAVs flying and hovering battery consumptions should be saved while maximizing the information gathered from the post-disaster area. 3) Finally, LEO-Sat has limited bandwidth resources mandating its optimal utilization based on UAVs traffic needs.

In this paper, we will address this problem using online learning by proposing a combinatorial multi-armed bandit (MAB) algorithm with fairness and UAVs battery budget restrictions for UAVs distribution [4]. Then and based on it, the gradient decent algorithm [5] will be used for optimizing the LEO-Sat bandwidth resources. Generally speaking, MAB [6] is an efficient reinforcement learning approach, where a player aims to maximize his achievable profit while playing over the bandit arms. The only available information to the player is the played arms along with their observed rewards, without any prior information about the game and its statistics. During the game, the player tries to balance between always exploiting the arm with the highest observable reward so far or exploring new ones, which is known as the *exploitation-exploration* tradeoff of the MAB games [7]. The proposed combinatorial MAB with fairness and budget restrictions is a type of MAB games, where the player selects a combination of multiple arms called the “super-arm” in each trial, while assuring fairness in the selected arms limited by the allowable cost budget paid by the player to select this “super-arm” [4]. In the proposed MAB model, the nearest survival GBS will act as the player, and the arms are the post-disaster zones over which UAVs should be distributed limited by their battery budget. Herein, the LEO-Sat will be used to relay data and control information between GBS and UAVs. To this end, the optimization problem will be formulated, and the combinatorial MAB with fairness and budget constraints (CMAB-FB) will be proposed to efficiently address the UAV distribution problem. Then, a gradient decent algorithm will be used to optimize the LEO-Sat bandwidth resources. Thus, the main contributions of this paper can be summarized as follows:

- An integrated LEO-Sat and UAVs network is proposed for rescue services in post-disaster area, where LEO-Sat will be used to relay control/traffic data to/from GBS from/to UAVs. In this network, the problem of UAVs distribution among users in this post-disaster area will be considered. The distribution of UAVs aims to maximize the total achievable data rate of the victims, while maintaining fairness in their coverage. Also, UAVs’ limited battery budget should be considered along with the limited bandwidth resources of the LEO-Sat. To the best of our knowledge, this is the first research effort for proposing LEO-Sat assisted UAV distributions in post-disaster scnearios.
- To efficiently address the efficient UAVs distribution problem within its UAVs energy constraints, it is considered as a MAB game, where “CMAB-FB” algorithm is proposed to implement it. The proposed “CMAB-FB” algorithm will be used to distribute the UAVs among the post-disaster zones to maximize the users’ achievable data rate while maintaining coverage fairness among them within the limited battery budget of UAVs. Then, based on the achieved UAVs rates, the bandwidth resources of the LEO-UAV links will be optimized subject to LEO-Sat limited bandwidth resources by means of gradient decent algorithm. By this way, the aforementioned challenges can be addressed as follows: 1) UEs coverage fairness can be assured based on their densities in post-disaster grids, where their densities can be pre-estimated using GPS localization and refined through UAVs exploration during the MAB game. 2) The budget constraint nature of the “CMAB-FB” algorithm results in saving UAVs energy resources. 3) The bandwidth resources of the LEO-Sat will be optimized based on the selected “super-arm”, i.e., grids, at each time slot.
- Numerical analyses are conducted to prove the effectiveness of the proposed scheme under different scenario. In this regard, the proposed scheme is compared against other benchmarks such as random and nearest UAV distribution, where the proposed scheme demonstrates superior performance over these benchmark schemes.

The rest of this paper is organized as follows: Section II gives the related works to that presented in this paper. Section III gives the proposed system model including the optimization problem formulation, Section IV gives the proposed “CMAB-FB” and the gradient descent algorithms, Section V gives the conducted numerical simulations, followed by the concluding remarks in Section VI.

## II. Literature review

Recently, extensive research has been undertaken to explore the utilization of UAVs for the purpose of supporting post-disaster areas. The synergistic employment of UAVs with cellular networks, and wireless sensor networks (WSNs) to facilitate disaster management applications was investigated in [8]. In another research endeavor given in [9], a genetic algorithm was employed to optimize the placement of UAVs, with the objective of enhancing both the overall coverage and data rate of the wireless network. In [10], an effective methodology to aid rescue operations in locating victims affected by a natural disaster was proposed. This approach involved the utilization of UAVs equipped with LiDAR and infrared depth cameras to construct an independent detection system that was not reliant on illumination intensity. Additionally, [11] involved the deployment of UAVs integrated with a video recorder and geolocation module, enabling the search for survivors within a post-disaster area. Furthermore, [12] explored the concept of flying communication services by equipping UAVs with Wi-Fi, video cameras, and web servers. The objective was to empower individuals affected by a disaster to utilize their smartphones for real-time text and video communication. Building upon this research, the authors of [13] proposed a mobility model based on the self-deployment of an aerial ad hoc network, utilizing the Jaccard dissimilarity metric to facilitate post-disaster scenarios. This mobility model incorporated the movement of victims and generated corresponding UAV mobility patterns to track these individuals. In the realm of disaster management systems, [14] presented a novel approach for energy-efficient task scheduling for the collected data obtained by UAVs from ground IoT networks. As for [15], UAVs were leveraged as on-demand airborne relays to establish connectivity between remote users and GBS, particularly when they were geographically isolated by substantial obstacles. In [16], millimeter wave (mmWave) UAV gateway selection is proposed in post-disaster scenarios. In [17], enhanced dynamic spectrum access was proposed empowered by MAB schemes for UAV networks in post-disaster scenario. This work was then extended in [18] to optimize the UAV 3D trajectory as well. Almost all work existing in literature regarding UAVs applications in post-disaster scenarios assumed that UAVs fly go-and-forth between the post-disaster zones and the ground fusion center in the nearest survival GBS. This will highly consume the UAVs energy and delay the rescue services operations. Moreover, all the above existing research works assumed a full awareness of the network parameters, which cannot be easily obtained in the completely destroyed infrastructure in post-disaster scenarios. In this paper, we leverage LEO-Sat as backhauling for the UAVs networks, which highly relaxes the need of UAVs flying between GBS and post-disaster zones. Moreover, as the proposed scheme is based on online learning, which does not need any prior information about the environment, except users’ pre-locations obtained using GPS services.

Regarding the integration between LEO-Sat and UAVs for enhancing/extending aerial coverage. In [19], the authors used a combination of LEO-Sat and UAVs for beyond-5G communication, utilizing millimeter-wave (mmWave) and free-space optical (FSO) links. A multi-agent deep reinforcement learning (MARL) approach is used to optimize communication and energy efficiencies, leading to improved peak and worst-case throughput compared to using only one of the links. In [20], the authors proposed the use of UAVs and LEO-Sat for data collection from internet-of-remote-things (IoRT) sensors. In [21], the authors proposed using LEO-Sat and caching by UAVs for content delivery in terrestrial networks to improve connectivity and capacity. The problem of optimizing cache placement, resource allocation, and trajectory was solved using an alternating algorithm. In [22], LEO-Sats were used for UAV tracking using Gauss Hermite filter based on hybrid TDOA/FDOA geolocation measurements. In [23], an integration between LEO-Sat and UAVs was considered for integrated mobile edge caching IoT system. In this model, LEO-Sat broadcasts data, and UAVs collect it from decentralized ground sensors. UAV deployment and power allocation for

secure space-air-ground communications was considered in [24]. The aim of the formulated optimization problem was to maximize the secrecy rate subject to UAV’s power and deployment area. Despite the existing research in LEO-UAV integration, none of them considered the problem of LEO-Sat assisted UAV distribution in post-disaster area as presented in this work.

### III. System model and optimization problem formulation

**Fig 1:**
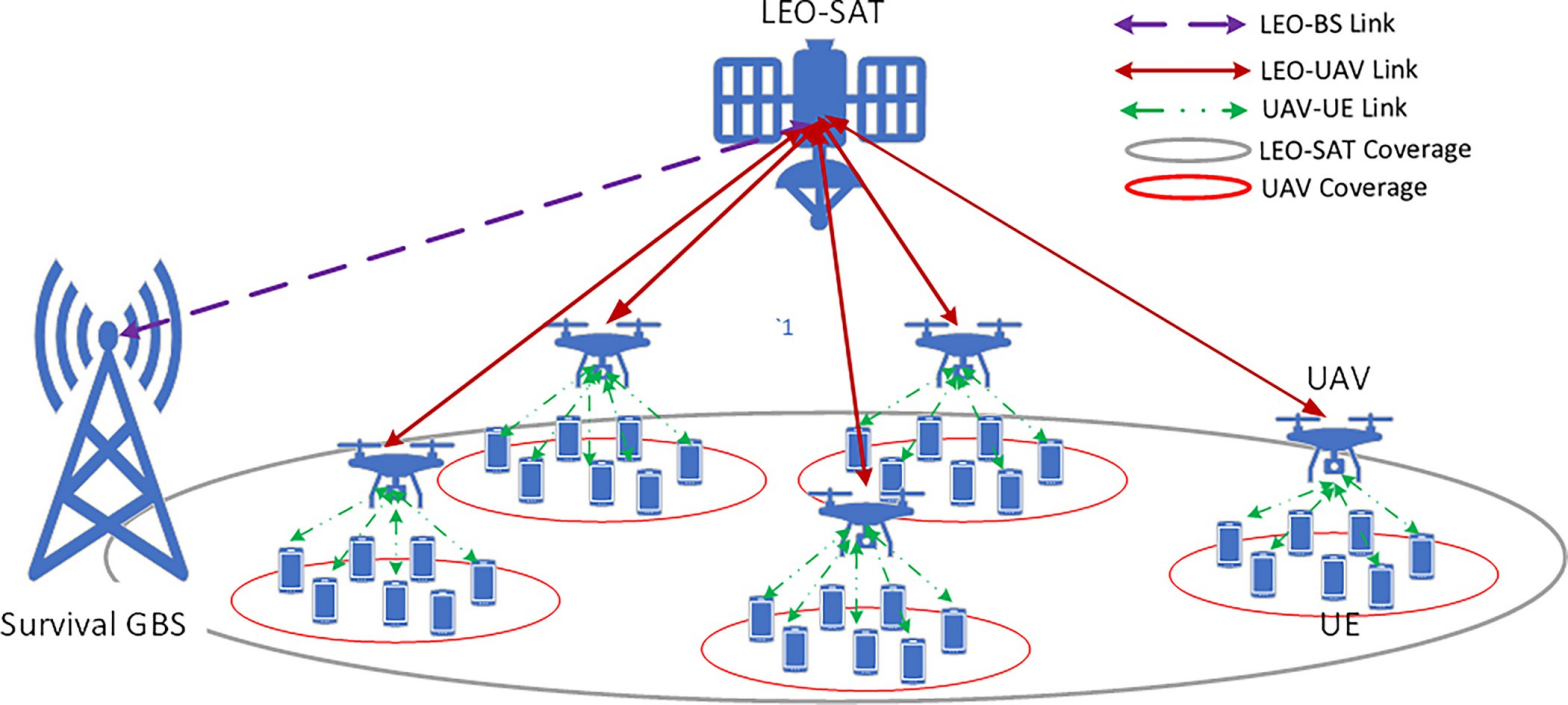
LEO-Sat assisted UAVs in a post-disaster area.

The proposed system model for LEO-UAV integration to cover the post-disaster area is shown in Fig 1. The area is divided into a set of *M* non-overlapped grids collected in ℳ. Each grid *i* ∈ ℳ contains *K*_*i*_ UEs. Dividing the post-disaster area into non-overlapped zones comes from the nature of the victims’ distributions which are typically formed in sparse groups due to the destruction happening in the area or grouping them within rescue shelters. This assumption was also considered in [25,26], when using UAVs in rescue services in post-disaster scenarios. In the proposed system model, LEO-Sat relays control and traffic data between GBS and UAVs for both control and traffic data. In this study, it is assumed that a set of *N* UAVs collected in N, *N* = 5 in Fig 1, where j∈N and *N*<*M*, are always within the coverage area of the LEO-Sat. At each time slot t, the GBS allocates the *N* UAVs to a selected group of the post-disaster grids, and it relays the information of the selected grids to the UAVs via the LEO-Sat. The criterion based on which the GBS selects the grids is to maximize the achievable data rates of their users, while assuring fairness in the grids coverage based on their users’ densities. Then, UAVs will fly towards the selected grides and hover above them to relay their users’ traffic data to the GBS via the LEO-Sat backhaul links. This operation is repeated over the time horizon constrained by UAVs’ battery capacities and LEO-Sat bandwidth resources. In the followings, the utilized UAV-UE and LEO-UAV link models will be explained in detail, and then the optimization problem of UAVs distribution will be formulated.

### A. UAV-UE link model

For the UAV-UE link model, we utilized the simple link model given in [27], which can be given as follows:

$$\Lambda_{{j,k}_{i}}(x_{{j,k}_{i}})=P^{LoS}\lambda_{{j,k}_{i}}^{LoS}(x_{{j,k}_{i}})+P^{NLoS}\lambda_{{j,k}_{i}}^{NLoS}(x_{{j,k}_{i}}),$$ (1)

$$\left\{ \begin{array}{c} \Lambda_{{j,k}_{i}}^{LoS}(x_{{j,k}_{i}})=20\;\log(\frac{4\pi x_{{j,k}_{i}}}{\lambda_{U}})+\rho^{LoS}\\ \Lambda_{{j,k}_{i}}^{NLoS}(x_{{j,k}_{i}})=20\;\log(\frac{4\pi x_{{j,k}_{i}}}{\lambda_{U}})+\rho^{NLoS} \end{array}\right.$$ (2)

$\Lambda_{{j,k}_{i}}(x_{{j,k}_{i}})$, $\Lambda (x_{{j,k}_{i}})$ and $\Lambda_{{j,k}_{i}}^{NLoS}(x_{{j,k}_{i}})$ are the total path loss, its line-of-sight (LoS) component, and non-LoS (NLoS) in dB between UAV *j* and UE *k* in grid *i* as a function of their separation distance $x_{{j,k}_{i}}$ Herein, *λ*_*U*_ is the wavelength of the UAV-UE link, and *ρ*^LoS^ and *ρ*^NLoS^ indicate the system loss in dB for both LoS and NLoS, respectively. ℙ^LoS^ and ℙ^NLoS^, where ℙ^NLOS^ = 1−ℙ^LOS^, are the probabilities for LoS and NLoS links. ℙ^LoS^ is defined as follows [27].

$$P^{LoS}={\left[1+aexp(-b(\theta_{j,k_{i}}-a))\right]}^{-1},$$ (3)

where *a* and *b* are constants based on the environment, while $\theta_{j{,k}_{i}}$ indicates the elevation angle between UAV *j* and UE *k* in grid *i*. Herein, $\theta_{j,k_{i}}=\tan^{-1}\left(\frac{h_{j}}{d_{{Hj,k}_{i}}}\right)$, where *h*_*j*_ is the hovering height of UAV *j* and $d_{{Hj,k}_{i}}$ is the horizontal distance between UAV *j* and UE *k* in grid *i*. Without loss of generality, uplink transmission is assumed between UEs in grid *i* and UAV *j*. Thus, the average data rate between UAV *j* and grid *i* at time slot t, $\Psi_{j,i}^{t}$, can be given as follows:

$$\Psi_{j,i}^{t}=\frac{B}{K_{i}}\sum_{k=1}^{K_{i}}{log}_{2}(1+P_{j,k_{i}}^{Rx,t}/\sigma^{2}),$$ (4)

In (4), frequency division multiple access (FDMA) is considered among UEs in grid *i*, where the total bandwidth *B* is equally divided among UEs in grid *i* which is denoted as *K*_*i*_. $P_{j,k_{i}}^{Rx,t}$ is Rx power at UAV *j* from UE *k* in grid *i* at time slot t, and *σ*^2^ is the noise power of the UAV-UE link, respectively. This model assumes uplink transmissions, but the same principle can be applied to the downlink. Moreover, the grids are assumed to be sparse as considered in [25,26], which prevents mutual interferences among grids, which is a reasonable assumption in post-disaster areas as previously explained.

### B. LEO-UAV link model

For LEO-UAV communication link, we utilized the link model given in [28], where the received power, $P_{S,j}^{Rx,t}$, and the achievable uplink data rate in bps at LEO-Sat from UAV *j*, $\eta_{S,j_{i}}^{t}$, at time slot t are written as follows:

$$P_{S,j}^{Rx,t}=\frac{P_{j,S}^{Tx}G_{j}^{Tx}G_{S}^{Rx}\lambda_{S}}{4\pi x_{S,j}^{t}},$$ (5)

$$\eta_{S,j}^{t}=B_{S,j}^{t}\log_{2}\left(1+\frac{P_{S,j}^{Rx,t}}{\tau \varepsilon B_{S,j}^{t}}\right),$$ (6)

where $P_{j,S}^{Tx}$ is the Tx power from UAV to LEO-Sat, and $G_{j}^{Tx},G_{S}^{Rx}$ are the Tx and Rx antenna gains from UAV (LEO-Sat) towards LEO-Sat (UAV), respectively. $x_{S,j}^{t}$ is the separation distance between the LEO-Sat and UAV. Also, *λ*_*S*_ is the wavelength of the LEO-UAV link. In (6), $B_{S,j}^{t}$, *τ* and *ε* are the allocated bandwidth of the LEO-Sat link at time slot t, the noise temperature, and the Boltzmann constant, respectively.

### C. Optimization problem formulation

At each time slot t, the GBS should decide which grids from ℳ the UAVs should cover, and then assigns UAVs to these selected grids. This information is communicated to the UAVs via the LEO-Sat. The objective is to distribute the UAVs-grids in a manner that maximizes the long term achievable data rates of the UAVs and assures fairness among the grids based on their UEs density, while also considering the limited battery capacities of the UAVs and the limited bandwidth of the LEO-Sat. Mathematically speaking, this optimization problem can be formulated as follows:

$$\underset{\eta_{S,j}^{t},d_{j,i}^{t}}{\max}\frac{1}{T}\sum_{t=1}^{T-1}\sum_{j=1}^{N}\sum_{i=1}^{M}d_{j,i}^{t}\;\min\left(\eta_{S,j}^{t},\Psi_{j,i}^{t}\right)$$ (7A)

s.t.

$$d_{j,i}^{t}\in \{0,1\}$$ (7B)

$$\sum_{j=1}^{N}\sum_{i=1}^{M}d_{j,i}^{t}\leq N$$ (7C)

$$\sum_{i=1}^{M}d_{j,i}^{t}=1\;\forall j\in N$$ (7D)

$$d_{j,i}^{t}\Gamma_{j,i}^{t}\leq \Gamma_{Umax}\;\forall j\in N$$ (7E)

$$\sum_{j=1}^{N}\sum_{i=1}^{M}d_{j,i}^{t}\eta_{S,j}^{t}\leq \eta_{Smax}$$ (7F)

$$\frac{1}{T}\sum_{t=0}^{T-1}\sum_{j=1}^{N}d_{j,i}^{t}\geq \frac{K_{i}}{\sum_{i=1}^{M}K_{i}}\;\forall i\in M$$ (7G)

where,

$$\Gamma_{j,i}^{t}=P_{f}T_{j,i}^{f,t}+P_{h}T_{j,i}^{h,t},$$ (8)

where *T* indicates the total time horizon, $d_{j,i}^{t}\in \{0,1\}$ is a selection indicator which is equal to one if grid *i* is selected to be covered by UAV *j* at time slot *t*, and zero otherwise. $\eta_{S,j}^{t}$ is the assigned capacity of the LEO-UAV of UAV *j* at time slot t. The 2^nd^ constraint in (7) means that the total number of selected grids should be less than or equal to the total number of UAVs *N* as the battery of some of UAVs may be completely depleted during the coverage process and needs for re-charging. The 3^rd^ constraint means that each UAV *j* should cover only one grid *i* at a time slot t. The 4^th^ constraint means that the energy consumption of UAV *j*, $\Gamma_{j,i}^{t}$, required to serve grid *i* at time slot *t* should not exceed its total battery capacity Γ_Umax_. $\Gamma_{j,i}^{t}$ is defined in (8) and considers both the flying and hovering powers (*P*_f_ and *P*_h_) and times $\left(T_{j,i}^{f,t}\;and\;T_{j,i}^{h,t}\right)$ of the UAV. $T_{j,i}^{f,t}$ is the ratio of the distance between the UAV’s current location and its target location in grid *i* at time slot *t*, divided by the UAV speed, while $T_{j,i}^{h,t}$ is determined by the traffic demand of grid *i* relative to $\Psi_{j,i}^{t}$. Actually, there are eight sources of UAV power consumptions as given in details in [29]. However, the flying and the hovering power consumptions are the most dominant ones as shown in [29], with flying consumes more energy than hovering [29]. Both *P*_f_ and *P*_h_ are related to the mass of the UAV, the gravitational force, the radius of the propeller, and the air density. In addition, *P*_f_ depends on the deviation angle between the UAV vertical axis and the *Z* axis as shown in [29]. For more details about various sources of UAV power consumption and their mathematical details, interested readers are advised to check [29]. The 5^th^ constraint means that as the link is established between UAV *j* and grid *i* at a time slot t, i.e., $d_{j,i}^{t}=1$. It should be assigned a bandwidth resource from the LEO-Sat with specific data rate $\eta_{S,j}^{t}$, where the sum data rates of all UAV-UE links corresponding to $d_{j,i}^{t}=1$ must not exceed the total capacity of the LEO-Sat, i.e., *η*_*S*max_. The 6^th^ constraint is used to ensure fairness among grides based on their UEs density.

### IV. Proposed CMAB-FB and gradient decent algorithms

The problem given in (5) is a dynamic non-linear programing problem, without a closed form optimal solution. However, this problem can be simplified by splitting it into two stages, where LEO-Sat capacity resources $\eta_{S,j}^{t}$ are typically optimized based on required UAVs traffic rates, i.e., $\Psi_{j,i}^{t}$, which is based on the optimized $d_{j,i}^{t}$ values. Thus, in the first stage, the UAV-grid association parameters $d_{j,i}^{t}$ are optimized, while in the second stage, $\eta_{S,j}^{t}$ values are adjusted based on the optimized $d_{j,i}^{t}$ and their corresponding $\Psi_{j,i}^{t}$. To optimize the values of $d_{j,i}^{t}$, the problem is considered as a combinatorial MAB problem with arms’ fairness constrained by UAV battery budget. Thus, in this section, we will explain the MAB model in general, then we introduce the proposed “CMAB-FB” algorithm for adjusting the values of $d_{j,i}^{t}$. Finally, the values of $\eta_{S,j}^{t}$ are adjusted using gradient decent algorithm.

### A. MAB model

MAB is an efficient online learning methodology, where a player plays over bandit arms and observes their rewards [7]. The player aims to maximize his long-term profit by learning to always play with the arm having the maximum achievable reward. The player has no-prior knowledge about the game except the played arms and their corresponding rewards. At each time slot during the game, the player tries to compromise between always exploiting the arm having the highest reward so far or exploring new unknown ones. This is what is called the *exploitation-exploration* dilemma of the MAB games [8]. There are several algorithms that can efficiently implement the MAB hypothesis like upper confidence bound (UCB), *ϵ*−greedy, Thompson sampling (TS), etc [30]. In some of the MAB games, selecting an arm comes with paying cost, which is constrained by the player’s limited budget. This type of MAB game is called budget constraints games, where budget constraint UCB and budget constraint TS are two well-known MAB algorithm variants that can efficiently implement these types of MAB games [31]. Also, in some cases, the player should select a group of arms from the arm space, which is called a “super-arm”; this type of MAB game is called a combinatorial bandit because a combination of arms should be selected at each time slot [32–34]. Sometimes fairness is required among the selected arms while selecting the super-arms, which are called combinatorial bandit with fairness constraints [4].

### B. Optimization of $d_{j,i}^{t}$

In this stage, we will optimize the values of $d_{j,i}^{t}$ adhering to constraints 1, 2, 3, 4 and 6. This is a time sequential combinatorial non-linear optimization problem, which can be viewed as a combinatorial MAB game with fairness considerations and UAV battery limitations. In this scenario, the player (i.e., GBS) chooses a “super-arm” by combining several “arms” (i.e., grids) at each time slot t, balancing the selection among the available arms based on their UEs density and minimizing the energy costs for the UAVs covering the chosen grids. Algorithm 1 gives the proposed “CMAB-FB” algorithm, where it is influenced by learning with fairness algorithm given in [4]. This algorithm and the LEO-Sat resource optimization will be run by the GBS platform as it is the player of the MAB game and the most powerful entity in the proposed LEO-UAV network. The inputs to the algorithm are the sets ℳ and N, and the design parameter Ω. Also, the values of UEs density $\delta_{i}=K_{i}/\sum_{i=1}^{M}K_{i}$ for grids is input to the algorithm, where *K*_*i*_ can be pre-estimated using GPS localization and then refined by UAVs exploration during the MAB game. For initialization, at t = 0, the selection vector, i.e., $w_{i}^{t}$, the number of times grid *i* was selected up to time slot t, i.e., $h_{i}^{t}$, the average rate of grid *i* up to time slot t, i.e., ${\hat{\gamma }}_{i}^{t}$, and the queue of grid *i*, $q_{i}^{t}$, are all set to zero ∀*i*∈ℳ. $q_{i}^{t}$ is used to assure fairness among the grids as will be explained shortly. For t = 1 to *T*, the upper confidence bound (UCB) values for ∀*i*∈ℳ are set to ${\bar{\gamma }}_{i}^{t}={\hat{\gamma }}_{i}^{t-1}+\sqrt{(3\;\log\;t)/(2h_{i}^{t-1})}$ if $h_{i}^{t-1}>0$ or ${\bar{\gamma }}_{i}^{t}=1$, if $h_{i}^{t-1}=0$. Then $q_{i}^{t}$ is evaluated for ∀*i*∈ℳ as given in the algorithm. At every time slot, the value of $q_{i}^{t}$ is increased by *δ*_*i*_ and decreased by 1 if grid *i* was selected in time slot *t*−1. Thus, if grid *i* is not selected many times, its $q_{i}^{t}$ is increased by multiples of its UE density value, and vice versa, which gives it a high priority for being selected in the next time slot. Thus, after evaluating $q_{i}^{t}$ and ${\bar{\gamma }}_{i}^{t}$ for ∀*i*∈ℳ, a super arm *A*(*t*)⊂ℳ is selected based on the following equation:

$$A^{t}=\underset{A^{t}\subset M}{\mathrm{argmax}}\sum_{i\in A^{t}}\left(q_{i}^{t}+\Omega {\bar{\gamma }}_{i}^{t}\right),|A^{t}|\leq N,$$ (9)

where Ω is a design parameter used to balance between selecting the grid maximizes the achievable average data rate or that maximizes fairness based on $q_{i}^{t}$ values. *A*^t^ can be easily evaluated by enumerating the |*A*^t^| grids having the highest values of $\left(q_{i}^{t}+\Omega {\bar{\gamma }}_{i}^{t}\right)$. After obtaining *A*^t^, the UAVs should be distributed among them, and obtaining $d_{j,i}^{*,t}$ in the way that minimizes their

Algorithm 1. Proposed CMAB-FB Algorithm.

Output: $d_{j,i}^{*,t}$

Input: M,N, initial values of *δ*_*i*_ using GPS localization, and Ω

Initialization: At *t* = 0, $w_{i}^{t}=0,h_{i}^{t}=0,{\hat{\gamma }}_{i}^{t}=0$, and $q_{i}^{t}=0\;\forall i\in M$

For *t* = 1,2,…,*T*

 For *i* = 1,2,…,*M*

  If $h_{i}^{t-1}>0$

    ${\bar{\gamma }}_{i}^{t}={\hat{\gamma }}_{i}^{t-1}+\sqrt{(3\;\log\;t)/(2h_{i}^{t-1})}$

   else

   ${\bar{\gamma }}_{i}^{t}=1$

    End if

  $q_{i}^{t}=max\{q_{i}^{t-1}+\delta_{i}-w_{i}^{t-1},0\}$

 End for

  • Select the super arm *A*^t^ as follows:

   $A^{t}=\underset{A^{t}\subset M}{\mathrm{argmax}}\sum_{i\in A^{t}}\left(q_{i}^{t}+\Omega {\bar{\gamma }}_{i}^{t}\right),|A^{t}|\leq N$

  • Select $d_{j,i}^{*,t}$ that minimizes UAVs energy consumptions as:

   1. Calculate $\Gamma_{j,i}^{t}$ matrix, ∀*i*∈*A*^t^ and ∀j∈N using (8)

   2. Connect UAV *j* to grid *i* as follows:

    For itr = 1:*N*

    a) $\{i^{*},j^{*}\}=arg\min\;\Gamma_{j,i}^{t}$

    b) $d_{j,i}^{*,t}=1$

    c) $\Gamma_{j^{*},i^{*}}^{t}=Inf$

   End for

  • Observe the average rates of the selected super arm *A*^t^ and update its related parameters as follows:

   1. $w_{i}^{t}=1\;\forall i\in A(t)$.

   2. $h_{i}^{t}=h_{i}^{t-1}+1\;\forall i\in A^{t}$

   3. ${\hat{\gamma }}_{i}^{t}=\frac{1}{h_{i}^{t}}\sum_{y=1}^{h_{i}^{t}}w_{i}^{y}\Psi_{j,i}^{y}\;\forall i\in A^{t}$

   4. Update *δ*_*i*_ ∀*i*∈*A*^t^

End for

energy consumptions as given in Algorithm 1. As a final step, the average data rate corresponding to the selected *A*^t^ are observed, and its related parameters are updated as given in the Algorithm 1 including the actual observed *δ*_*i*_ values corresponding to *A*^t^. As the “super-arm” selection in the proposed “CMAB-FB” algorithm is done in the same way as that given in [4]. The time average accumulative regret of the proposed algorithm is the same, which is defined as follows [4]:

$$R(T)\leq \frac{M}{2\Omega }+\frac{2\sqrt{6NMTlogT}+(1+\frac{5\pi^{2}}{12})M}{T},$$ (10)

For detailed derivation of (10), readers are advised to check [4].

### C. Optimization of $\eta_{S,j}^{t}$

In the second step after obtaining $d_{j,i}^{*,t}$, the term $\Psi_{j,i}^{t}$ becomes a constant in (7), then we can optimize the values of $\eta_{S,j}^{t}$ under constraint 5 as follows:

$$\underset{\eta_{S,j}^{t}}{\max}\frac{1}{T}\sum_{t=1}^{T-1}\sum_{j=1}^{N}\min\left(\eta_{S,j}^{t},\Psi_{j,i}^{t}\right)$$ (11)

s.t.


$$\sum_{j=1}^{N}\eta_{S,j}^{t}\leq \eta_{Smax}$$

Typically, the goal of optimizing LEO-Sat resources $\eta_{S,j}^{t}$ is to accommodate the traffic demands of UAVs $\Psi_{j,i}^{t}$ within LEO-Sat maximum capacity constraint *η*_*S*max_. This allows us to simplify the optimization problem given in (11) as to minimize the absolute difference between LEO-Sat offering rate $\eta_{S,j}^{t}$ and $\Psi_{j,i}^{t}$ as follows:

$$\underset{\eta_{S,j}^{t}}{\min}\frac{1}{T}\sum_{t=1}^{T-1}\sum_{j=1}^{N}abs|\eta_{S,j}^{t}-\Psi_{j,i}^{t}|$$ (12)

This problem is a non-linear programing problem due to the absolute value. However, it can be linearized by simplifying the absolute function, then solved by any famous iterative method like gradient decent method as given in Algorithm 2. In this Algorithm, we omit t for notation simplification. The inputs to the algorithm are Ψ_*j*,*i*_ obtained after UAVs distribution done by Algorithm 1, *η*_*S*max_, the value of the learning rate *α*, the maximum number of iterations *MaxIter*, the tolerance value *Tolernc*, and the number of UAVs *N*. The output is the optimized values of $\eta_{S,j}^{*t}$. For initialization, the values of *η*_*S*,*j*_ are randomly selected. Then for *iter* = 1 to *MaxIter*, the gradient vector, *Gradient*, of size1×*N* is set to 0. Then for *j* = 1 to *N* the difference between *η*_*S*,*j*_ and Ψ_*j*,*i*_ is calculated, if it is greater than or equal to 0, the *Gradient* {*j*} is set to +1, otherwise it is set to -1. Then, the values of *η*_*Sj*_ are updated as follows:

$$\eta_{S,j}=\eta_{S,j}-\alpha \times Gradient,\;\forall j\in N,$$ (13)

After updating *η*_*S*,*j*_, the conditions of maximum capacity constraint, the gradient tolerance and the maximum number of iterations are tested successively and if any one of them is satisfied, the value of $\eta_{S,j}^{*}$ is returned and the Algorithm will be terminated. After adjusting the values of $\eta_{S,j}^{*t}$, the values of $B_{S,j}^{*t}$ can be easily obtained by solving (6).

From Algorithm 1 and 2, we can conclude that the fairness, UAVs energy conserving and LEO-Sat resource optimization provided by the proposed scheme comes at the expense of slight increase in computational complexity compared to other naïve benchmarks.

Algorithm 2. Gradient Decent Algorithm for $\eta_{S,j}^{t}$ Optimization.

Output: $\eta_{S,j}^{*}$

Input: Ψ_*j*,*i*,_ *η*_*S*max_, *α*, *MaxIter*, *Tolernc*, *N*

Initialization: *η*_*S*,*j*_ is randomly initialized

For *iter* = 1,2,…,*MaxIter*

   *Gradient*{1:*N*} = 0

  For *j* = 1,…,*N*

   If *η*_*S*,*j*_−*Ψ*_*j*,*i*_≥0

     *Gradient*{*j*} = +1

   else

     *Gradient*{*j*} = −1

   End if

  End for

  $\eta_{S,j}=\eta_{S,j}-\alpha \times Gradient\;\forall j\in N$

   $\sum_{j=1}^{N}\eta_{S,j}\leq \eta_{Smax}$

    $\eta_{S,j}^{*}=\eta_{S,j}$

   Break For

  nd If

  If ‖*Gradient*‖_2_≤*Tolernc*

    $\eta_{S,j}^{*}=\eta_{S,j}$

   Break For

  End If

  If *iter* = *MaxIter*

   $\eta_{S,j}^{*}=\eta_{S,j}$

  End If

End for

## V. Numerical analysis

In this section, the effectiveness of the proposed “CMAB- FB” algorithm is evaluated through comprehensive Monto-Carlo

simulations. A post-disaster area of 1 Km^2^ is considered, which is divided into 36 grids, each with a varying number of users. The altitude of the LEO-Sat is set at 550 kilometers, and the UAV are flying at 100 meters in height. It is assumed that the LEO-Sat has full coverage of the post-disaster area. The Tx power of the LEO-Sat is set to 10 watts, while the Tx power of the UAV and UE are set to 1 watt. The total bandwidth available to the LEO-Sat is 100 MHz, and the bandwidth assigned to the UAV is 40 MHz. Additional simulation parameters are listed in Table 1.

**Table 1:** Simulation parameters.

Parameter	Value
*η*_*S*max_, Γ_Umax_	100 MHz [28], 10 KJ
*K*_*i*_	Uniformly random in the range [1,100]
*f*_*U*_, *f*_*S*_	2 GHz [27], 2.4 GHz [28]
*σ*^2^(dBm)	-174 + 10log_10_(*W*) + 10 [35]
*P*_*f*_, *P*_*h*_	4, 2 Watt [16] [25]
*τ* and *ε*	1000 and 1.38×10^−23^ [28]
*ρ*^LOS^ and *ρ*^NLOS^	0.1 dB and 21 dB [27]
UE data load	5 Gbit
*T*, Ω	1000, 0.01
$G_{j_{i}}^{Tx}G_{S}^{Rx}$	15 dB [28]
*a*, *b*	4.88, 0.429 [27]

As there are no comparable schemes exist in literature that address the same problem, the performance of the proposed “CMAB-FB” algorithm is compared with two naïve benchmarks: random “Rand” selection and nearest “Nearest” selection. In the random selection method, grids are randomly chosen by GBS with the constraint that each UAV can only serve one grid at a time. The LEO-Sat bandwidth $B_{S,j}^{*t}$ is also randomly distributed across [1, total Sat bandwidth/*N*]. In the nearest selection method, the GBS chooses the closest grid for each UAV to serve, with the same constraint that each UAV can only serve one grid at a time. Moreover, the LEO-Sat bandwidth $B_{S,j}^{t}$ is equally divided among UAVs.

**Fig 2:**
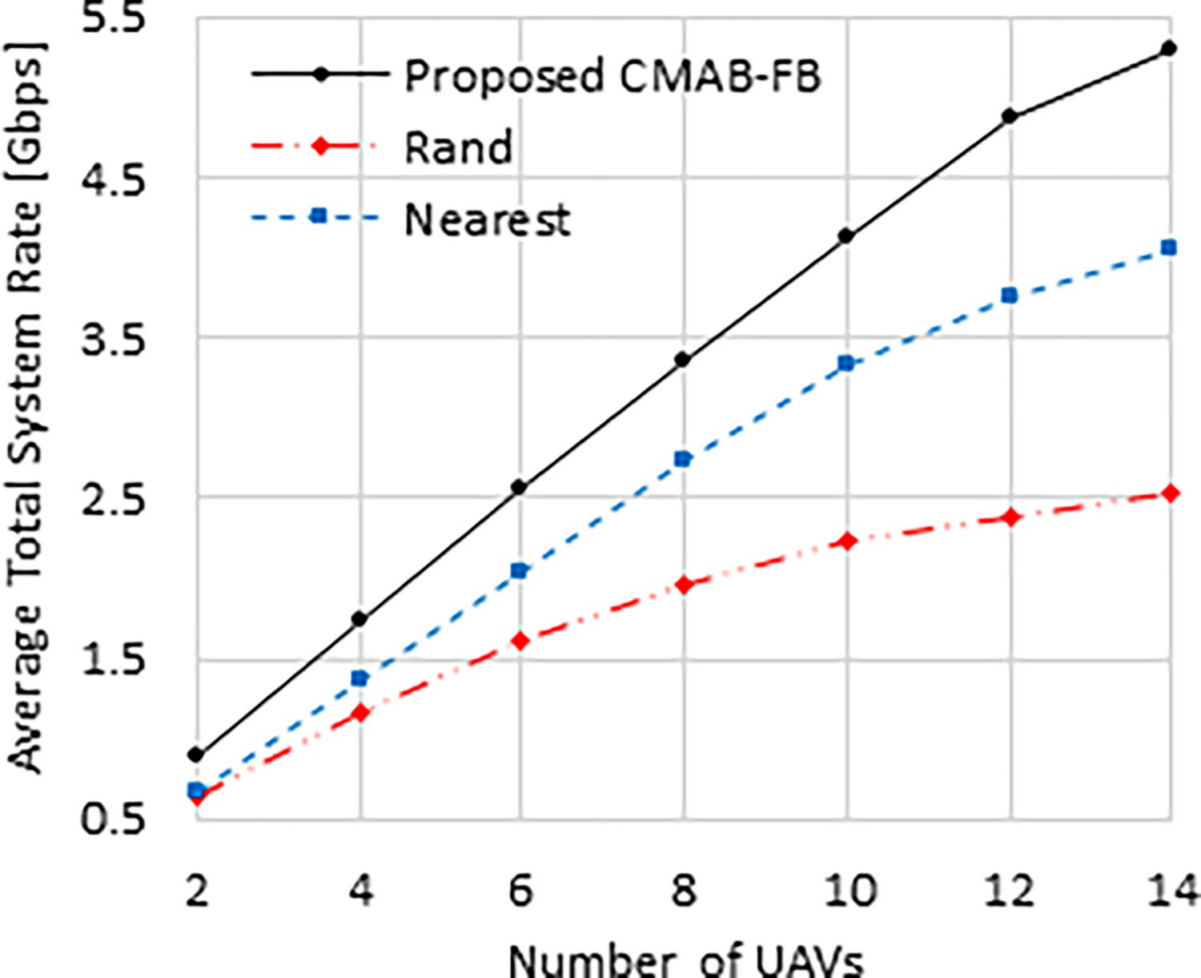
Average total system rate against number of UAVs.

Fig 2 shows the average total system rate in Gbps against the number of UAVs. The total system rates of all compared schemes are increased as we increase the number of UAVs due to covering more grids at a time. The proposed “CMAB-FB” scheme has the best performance among the compared schemes due to its objective of maximizing the sum of average UAVs rates at each time step. Additionally, it is noteworthy that the “Nearest” scheme has better performance compared to the “Rand” scheme, because in the nearest selection, the LEO-Sat bandwidth is shared equally among the UAVs, whereas in the random selection, the LEO-Sat bandwidth is randomly assigned to each LEO-UAV link. At *N* = 2, the proposed “CMAB-FB” scheme outperforms “Nearest” and “Rand” schemes by 1.327 and 1.5 times, respectively. These values become 1.32 and 2 times at *N* = 14, respectively.

**Fig 3:**
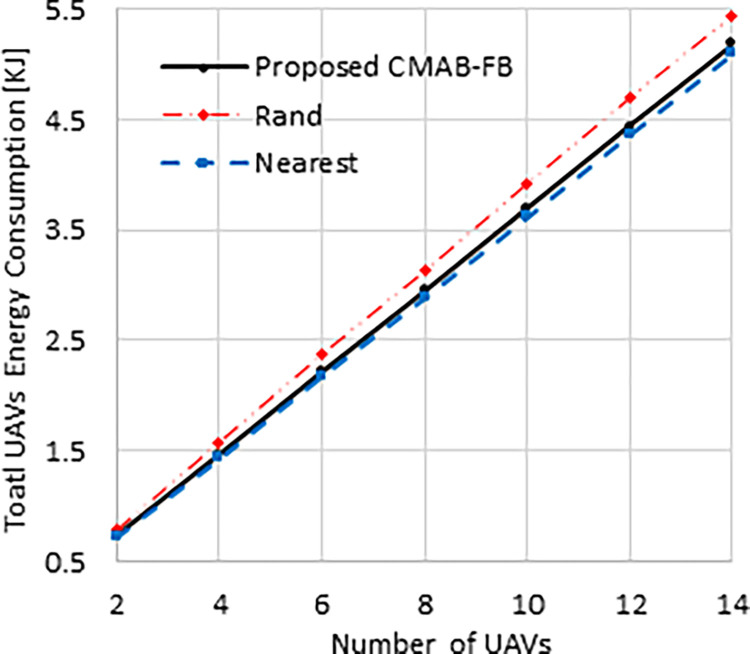
Energy consumption against number of UAVs.

Fig 3 illustrates the relationship between the number of UAVs and the average total energy consumption of UAVs. According to this figure, the “Rand” method has the highest energy consumption performance due to its random grid selection. The “Nearest” method, on the other hand, consistently chooses the closest grids to the UAVs and thus has the lowest energy consumption performance. The proposed “CMAB-FB” method, with its focus on UAVs energy consumption minimization, performs similarly

to the “Nearest” method. It is worth mentioning that all the methods have comparable energy efficiency performance due to the constraint that only one UAV can cover one grid at a time. This means that a UAV may have to choose its second or another nearest grid if its closest grid is already being covered by another UAV. Despite this constraint, the proposed “CMAB-FB” method still has performance comparable to the “Nearest” method. At *N*= 14, both the “CMAB-FB” and the “Nearest” schemes show better energy efficiency performance than the “Rand” method by 5% and 7%, respectively.

**Fig 4:**
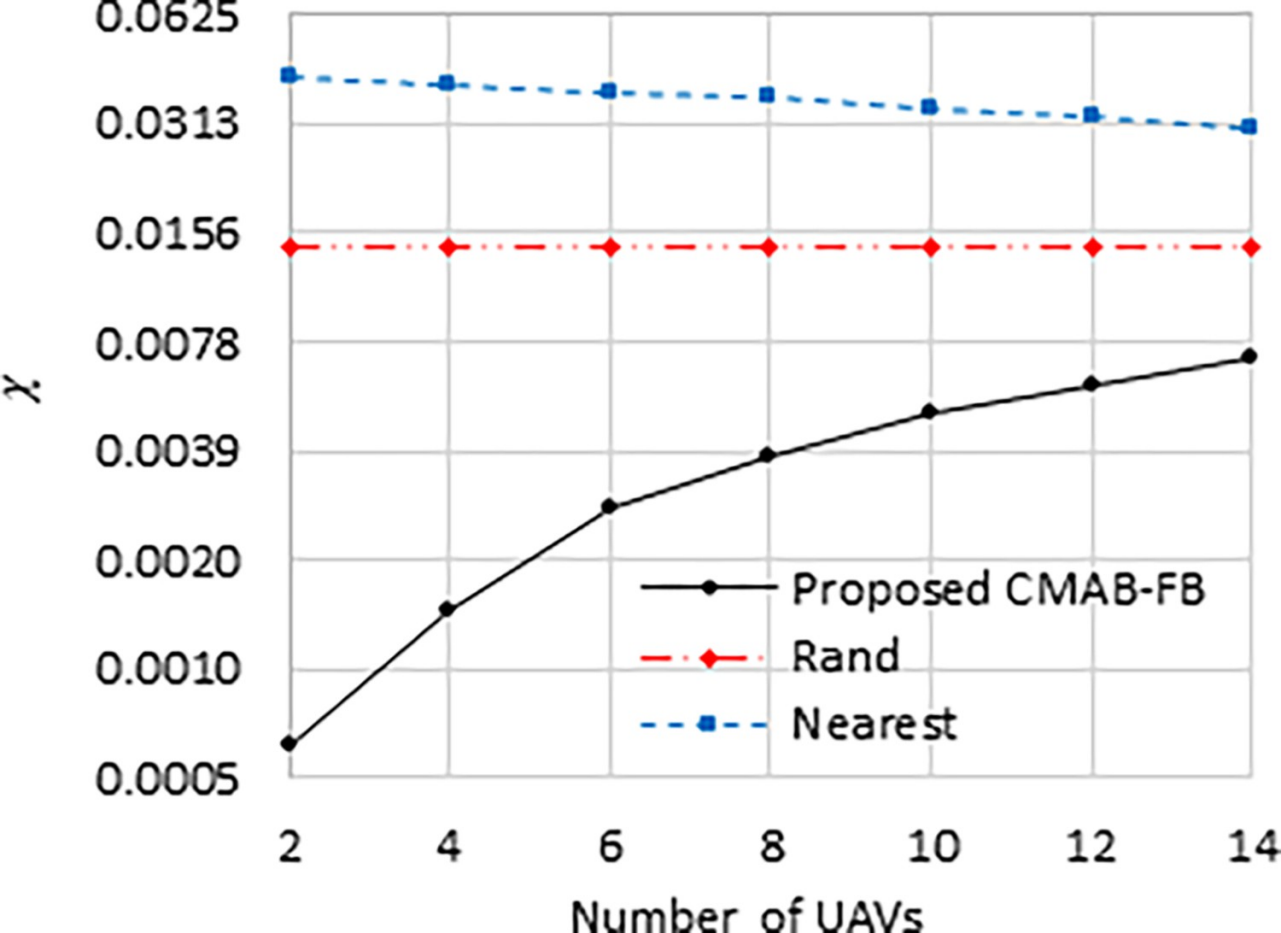
Grids selection fairness index against number of UAVs.

Fig 4 displays the fairness index in grids selection, which is calculated as:

$$\chi =\sum_{i=1}^{M}abs|\frac{h_{i}^{T}}{\sum_{i=1}^{M}h_{i}^{T}}-\delta_{i}|,$$ (14)

where the term $\left(\frac{h_{i}^{T}h_{i}(T)}{\sum_{i=1}^{M}h_{i}^{T}h_{i}(T)}\right)$ indicates the number of times grid *i* is selected relative to the total number of grids selections. A value of *χ* close to 0 indicates that grids are selected based on their UE densities, as determined by the value of *δ*_*i*_. From Fig 4.

The proposed “CMAB-FB” scheme has the lowest values of *χ*, which demonstrates its ability to distribute UAVs over grids fairly based on their UEs density. The “Nearest” scheme has the worst fairness performance as it always selects the nearest grids, while “Rand” scheme has better fairness performance than “Nearest” scheme as it selects grids uniformly. This is the reason why *χ* remains constant, regardless of the number of UAVs tested, in the “Rand” scheme. However, using “Nearest” scheme, *χ* tends to decrease as we increase the number of UAVs because many UAVs will be better distributed among their nearest available grids. Also, as the number of UAVs grows, the *χ* values of all schemes tend to become more like one another due to the reduced number of grid groups available for selection at each iteration. At *N* = 2, the proposed “CMAB-FB” scheme has lower *χ* values than “Nearest” and “Rand” schemes by 70 and 24 times, respectively. These values become 4.2 and 2.14 at *N* = 14, respectively.

**Fig 5:**
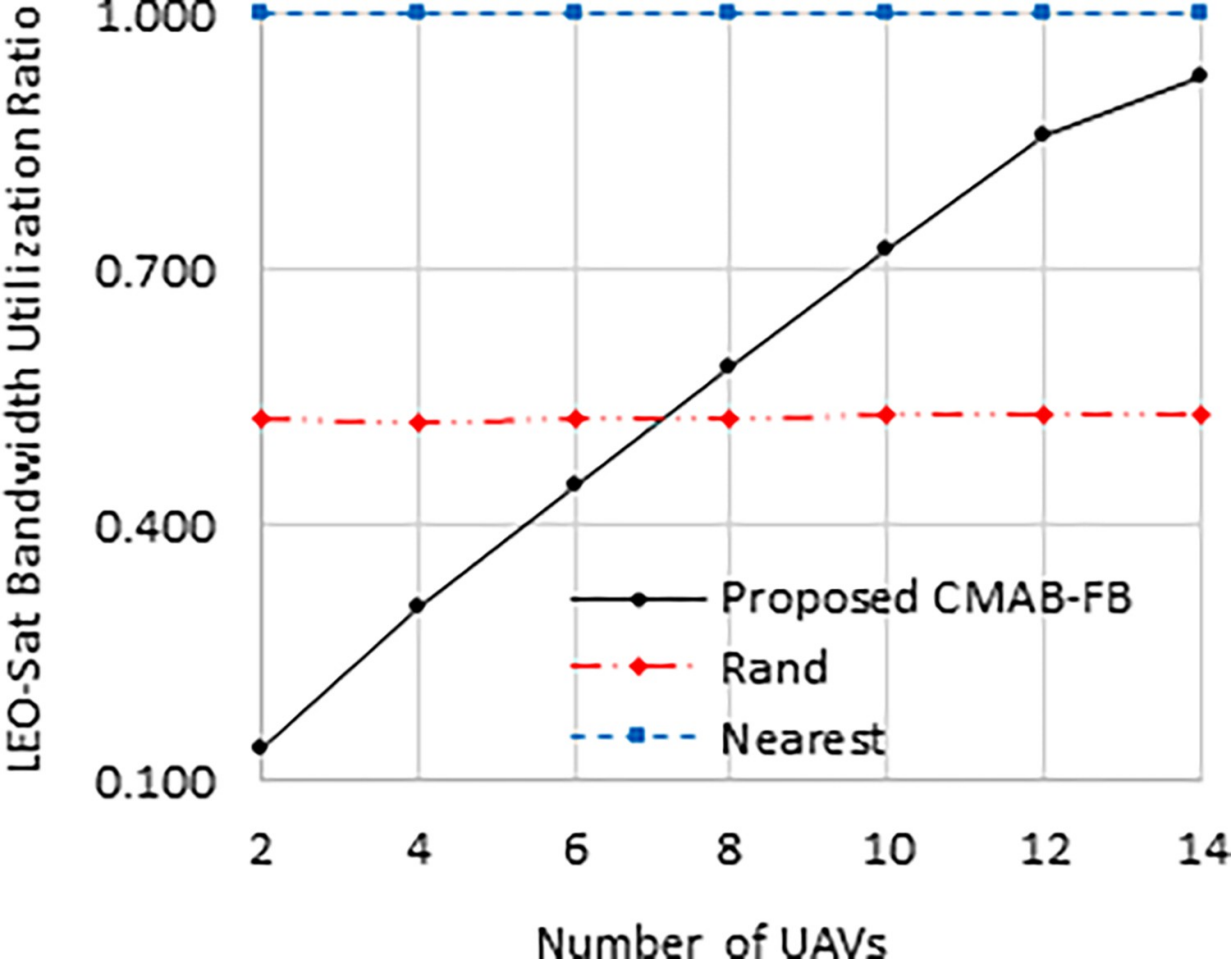
LEO-Sat bandwidth utilization ratio against number of UAVs.

The utilization ratio of the LEO-SAT bandwidth in the compared schemes is displayed in Fig 5. This figure demonstrates that the proposed “CMAB-FB” scheme optimizes the LEO-Sat bandwidth utilization. With a low number of UAVs, only a small portion of the LEO-Sat bandwidth is used relative to their traffic needs, but with a high number of UAVs, a larger portion of the LEO-Sat bandwidth is utilized. The “Nearest” scheme has a fixed utilization ratio of one, as the LEO-Sat bandwidth is equally divided among UAVs, regardless of their number or traffic needs. The “Rand” scheme has a fixed utilization of 0.5, as the bandwidth is uniformly distributed in the range [1, total Sat bandwidth/*N*]. With a high increase in the number of UAVs, the proposed “CMAB-FB” scheme approaches a utilization ratio of one, as the UAVs’ traffic needs require the full utilization of the LEO-Sat bandwidth.

The computational complexity of the proposed “CMAB-FB” scheme composed of three components. The first one comes from minimizing energy consumption with computational complexity of $O(N^{2})$, the second one comes from the sorting operation with computational complexity of O(N), and the third one comes from the gradient decent algorithm used to optimize LEO-Sat bandwidth resources with computational complexity of O(N). Therefore, the computational complexity of the proposed algorithm mainly depends on the square of the number of UAVs. Compared to the other benchmarks, the computational complexity of the rand selection is of order O(N) as it will generates *N* random numbers in the range of [1, *M*]. Also, the computational complexity of the nearest selection is of order O(N) as each UAV will select its nearest grid.

## VI. Conclusion

In conclusion, this study has focused on the distribution of UAVs in a post-disaster area with the help of LEO-Sat. The goal was to achieve a balance between maximizing the total sum rate of UAVs and ensuring fairness in the coverage of post-disaster grids based on their UEs density. This is done subject to the limited energy and bandwidth resources of UAVs and LEO-Sat. To address this challenge, we have deemed it as a combinatorial MAB with fairness and budget constraints, and we have proposed the “CMAB-FB” algorithm to solve it efficiently. The study also has proposed a way to optimize the LEO-Sat bandwidth based on UAVs’ traffic needs. The results of the numerical analysis have demonstrated that the proposed “CMAB-FB” scheme has outperformed other naïve benchmark approaches.
